# Successful treatment of refractory systemic lupus erythematosus-associated immune thrombocytopenia with drug-induced liver injury with telitacicept: a case report and review

**DOI:** 10.3389/fimmu.2025.1473190

**Published:** 2025-03-19

**Authors:** Xuefei Li, Yao Fu, HaiQin Yin, Huiling Zhu

**Affiliations:** Department of Rheumatology and Immunology, Jiujiang University Affiliated Hospital, Jiujiang, Jiangxi, China

**Keywords:** immune thrombocytopenia, systemic lupus erythematosus, drug induced liver injury, telitacicept, refractory

## Abstract

Immune thrombocytopenia(ITP)is a common clinical manifestation of systemic lupus erythematosus(SLE). Drug therapy includes glucocorticoids(GCs),disease-modifying anti-rheumatic drugs (DMARDs) and biologics. Refractory thrombocytopenia can be life-threatening, and the use of effective medications plays a crucial role in disease improvement. Here, we report a case of ITP secondary to SLE. The use of dexamethasone(DEX), cyclosporine A(CsA), and hetrombopag resulted in drug-induced liver injury. Subsequently, telitacicept was chosen and successfully controlled the patient’s condition. It suggests that telitacicept may be a new treatment option for refractory SLE-ITP.

## Introduction

Systemic lupus erythematosus (SLE) is a chronic autoimmune disease that can cause damage to multiple organs, with hematological involvement being a common systemic manifestation. Studies have shown that B-cell hyperactivity and the production of anti-platelet-associated antibodies are the main mechanisms of immune thrombocytopenia(ITP) secondary to SLE (1). Conventional treatments are based on glucocorticoids(GCs) and immunologic agents, among others. Here, we describe a case of an SLE-ITP patient, who developed hepatic impairment after various medications treatments and was successfully treated with a combination of GCs and telitacicept. The relevant literatures are discussed to reveal the disease characteristics and potential therapeutic targets of SLE-ITP.

## Literature review

We searched the literature through major scientific databases, including PubMed, ScienceDirect, SpringerLink, and Web of Science,and using the keywords:”telitacicept”. We found only 12 case reports articles reporting telitacicept treatment. The search results are shown in **Table 1**.

**Table 1:** Characteristics of 13 patients with autoimmune diseases treated with telitacicept.

Authors	Time	Sex	Age	Diagnoses	Symptoms	Prior treatments Treatment	Improvement
Ma et al. (2)	2022	female	39	SLE	alopecia areata	GCs+HCQ+LEF+MTX+MMF	Yes
Zhang et al. (3)	2022	male	46	MN	nephropathy	GCs+Tac+MMF+CYC+RTX	Yes
Chen et al. (4)	2022	female	33	SLE	Proliferative lupus nephritis	GCs+HCQ+TGP+MMF	Yes
Huang et al. (5)	2023	male	64	GPA	rapidly progressive glomerulonephritis	GCs+IVIG+CYC	Yes
Wang et al. (6)	2023	male	32	MN(stage I-II)	nephropathy	Tac+RTX	Yes
Guo et al. (7)	2023	female	75	MG	ptosis, dysphagia and limb weakness	GCs+IVIG+Tac	Yes
Ren et al. (8)	2023	female	14	NF155+ AN	muscle weakness, numbness of distal limbs, dysphagia, and slurred speech	GCs+RTX+PE+IA+MMF	Yes
Tian et al. (9)	2023	female	23	SLE+MOG-AD	difficulty urinating, headache, unsteady walking, numbness in the limbs and body	GCs+IVIG+MMF+HCQ	Yes
Li et al. (10)	2024	female	25	SLE	nephritis	GCs+MMF	Yes
Huang et al. (11)	2024	female	35	SLE	LN+recurrent leukopenia	GCs+MMF+HCQ+SIR+Belimumab	Yes
Zhang et al. (12)	2024	male	64	MG	bilateral ptosis	GCs+IVIG+Tac	Yes
		female	70	MG+RA	ptosis, dysarthria, and dysphagia	IVIG	Yes
Fan et al. (13)	2024	female	23	SLE	Lupus hepatitis	GCs+MMF+HCQ+AZA+Belimumab	Yes

SLE, systemic lupus erythematosus; MN, membranous nephropathy; GPA, Granulomatous polyangiitis; MG, myasthenia gravis; NF155+AN, neurofascin-155+autoimmune nodopathy; MOG-AD, myelin oligodendrocyte glycoprotein antibody disease; RA, rheumatoid arthritis; GCs, glucocorticoids; HCQ, hydroxychloroquine; LEF, leflunomide; MTX, methotrexate; MMF, mycophenolate mofetil; Tac, tacrolimus; CYC, cyclophosphamide; RTX, rituximab;TGP, total glucosides of paeony; IVIG, intravenous immunoglobulin; PE, plasma exchange; IA, immunoadsorption; SIR, sirolimus; AZA, azathioprine.

## Case report

On June 3, 2023, a 31-year-old female patient with cyanotic skin and ecchymosis for three days was admitted to the hematology department. The patient had no special previous medical history. Laboratory tests showed platelet(PLT) counts of 4×10^9/L, positive anti-nuclear antibodies (ANA) (1:1000 granular type),positive anti-SSA/Ro60 antibodies, positive anti-u1-SnRNP antibodies. Liver and kidney function, complement, immunoglobulin, rheumatoid factor, haemagglutination function, AT-III activity test, thromboelastography test, antiphospholipid antibody, anti-ADAMTS13 antibody, and antiplatelet antibody were normal. Bone marrow (BM) cytology revealed increased nucleated cells, impaired maturation of megakaryocytes (MKs), and decreased PLTs.Treatment included PLT transfusion, subcutaneous injection of recombinant human thrombopoietin (TPO), oral dexamethasone (DEX; 40mg/day for 3 days), and intravenous immunoglobulin (IVIG) (20g/day for 2 days).

After treatment, the patient’s PLT counts were rechecked at 86×10^9/L on June 8th. Then, she was transferred to the Department of Rheumatology and Immunology for further treatment, considering thrombocytopenia secondary to connective tissue disease.After transferring to our department, she received methylprednisolone (MP) 40mg qd and hydroxychloroquine (HCQ) 0.2g bid. On June 15, the PLT counts were normal, but alanine aminotransferase (ALT) and aspartate aminotransferase (AST) were significantly elevated (ALT471.70U/L, AST90.72U/L).Analyzing the cause of acute hepatic impairment, the possibility of drug induced liver injury was deemed high. Upon reviewing the patient’s medications, it was discovered that oral DEX 40mg for 3 days was likely the cause of the hepatic damage. As a result, other suspected medications were discontinued, and only MP along with basic hepatoprotective drugs (magnesium isoglycyrrhizinate 200 mg/day and glutathione 1800mg/day) were continued.On July 3rd, the liver function returned to normal, but the PLT count decreased again to 6×10^9/L. Therefore, MP was given to increase the dosage to 200mg qd, and interleukin-11, PLT transfusion and IVIG 10g infusion were also given.The next day, hetrombopag (2.5mg qd) and CsA (50mg bid) orally were added.

**Figure 1:**
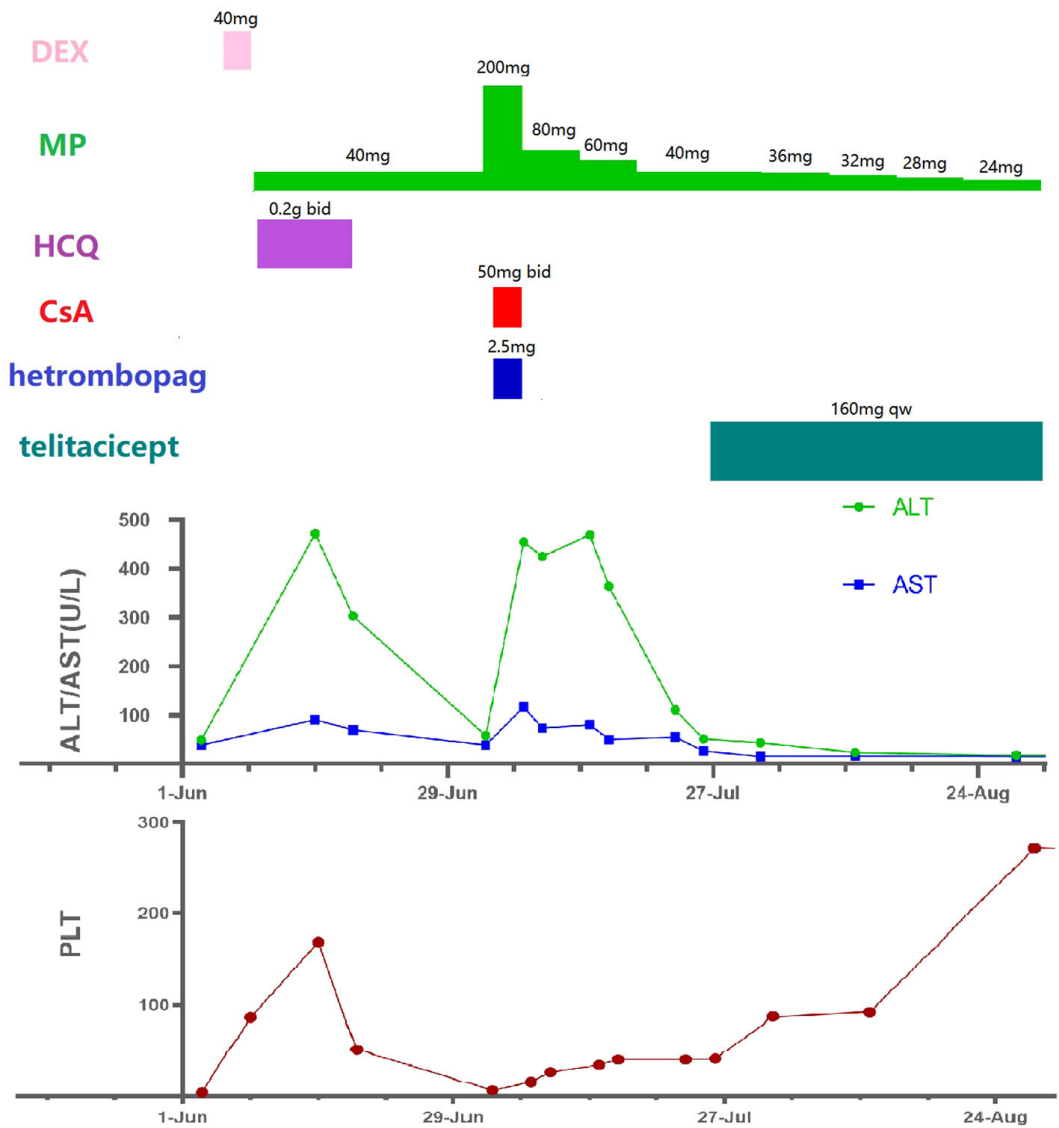
Medication process and laboratory indicators(all timelines are the same). DEX, dexamethasone; MP, methylprednisolone; HCQ, hydroxychloroquine; CsA, cyclosporine A; ALT, alanine aminotransferase; AST, aspartate aminotransferase; PLT, platelet.

On July 7, the PLT counts were repeated at 15 x 10^9/L, however, ALT and AST were again elevated (ALT 454.60 U/L, AST 116.91 U/L).After further exclusion of autoimmune liver disease and viral hepatitis, the patient was evaluated for another short-term sharp increase in liver enzymes, and drug induced liver injury was still suspected. The decision was made to stop CsA and hetrombopag, enhance liver protection and enzyme-lowering therapy, and continue the treatment of MP (80mg qd) and interleukin-11.Considering the patient’s recurrent PLT decrease, despite the effectiveness of GCs and IVIG treatments, severe liver damage occurred when CsA, hetrombopag and other drugs were added. When the GCs was slightly reduced, there was a significant drop in PLTs. In light of the patient’s hepatic impairment from multiple drugs, it was decided to refrain from adding additional conventional disease-modifying antirheumatic drugs (cDMARDs) at this time. Therefore, it was recommended that the patient try using telitacicept. After she agreed, screening was conducted to exclude infectious diseases. On July 26th, PLT counts of 41 x 10^9/L and normal liver function (ALT 51.26 U/L, AST 26.60 U/L)were rechecked before treatment. On July 27th she was started on telitacicept 160 mg subcutaneously and on August 1st she was retested for PLTs 87 x 10^9/L, ALT 43.52 U/L,AST 15.49 U/L.She continued regular telitacicept 160 mg once weekly in combination with MP orally.on August 28, the PLT counts were repeated at 272 x 10^9/L and liver function was normal (**Figure 1**).

In December, the patient discontinued MP and telitacicept on her own. Half a month later, she developed swelling and pain in both elbows and ankles, and a facial rash, which fulfilled the 2019 EULAR/ACR classification criteria for SLE (14), and led to the diagnosis of SLE. On January 18, the patient resumed the use of MP 8mg qd, HCQ 0.2g bid, and telitacicept 160mgqw, with symptomatic relief. On February 25, PLT204×10^9/L was rechecked, and liver and renal functions were normal.

## Discussion

In patients with SLE,the immune response mediates dysregulation of PLT production and destruction, which in turn leads to ITP (15). Lupus patients can produce a large number of antiplatelet-related antibodies, including anti-GPIbα, anti-gpia/IIa, anti-gpIIb/IIIa, anti-PAIgG, anti-gpib/IX, anti-CD40L antibodies, antiphospholipid antibodies, anti-c-mpl and anti-TPO antibodies (1). The cause of excessive antibody production may be due to the high expression of the stimulatory factors B-cell activation factor (BAFF) and a proliferation-inducing ligand (APRIL), leading to overactivation of B cells (16), and increased autoantibodies on PLT membranes caused by autoimmune B cell clearance disorders. BAFF (also known as B-lymphocyte stimulator,BLyS)and APRIL are members of the tumor necrosis factor (TNF) family (17). They share two receptors, transmembrane activator or calcium-modulating cyclophilin ligand-interactor (TACI) and B cell maturation antigen (BCMA), and BLyS can also bind to a third receptor, B cell activating factor receptor (BAFFR) (18). The process of BAFF/APRIL binding to its receptor is crucial for the proliferation, differentiation and maturation of B cells.It also influences the formation of live and long-lived plasma cells in the spleen (19). PLT antigens in the spleen are mainly delivered to T helper (Th) cells by dendritic cells (DCs)and macrophages, causing increased secretion of BAFF, This further operates through receptors to induce typical and atypical NF-κB activation, which in turn leads to B-cell maturation.The typical NF-κB pathway is activated by TACI receptors, and the sustained expression of Blimp-1 by B cells leads that B cells differentiate into autoantibodies-secreting plasma cells (20) and long-lived plasma cells (21), which causes the secretion of large amounts of pathologic IgG by autoreactive B cells.Furthermore, the high expression of BAFF in the sera of patients with active SLE induces an increase in the proportion of immature regulatory B cells (Bregs), which leads to a decrease in the proportion of mature Bregs subpopulations, thereby exacerbating disease activity (22).

The main drugs used in the clinic for the treatment of SLE-ITP are GCs, IVIG, cDMARDs, biologic agents and thrombopoietin-receptor agonists. For refractory thrombocytopenia, biologics like rituximab (RTX) are also a viable choice. In pharmacology, RTX depletes CD20-expressing B cells instead of Pro-B cells or plasma cells. It has been found that B-cell depletion leads to increased levels of BAFF in the spleen and serum, which contributes to plasma cell survival and differentiation into long-lived plasma cells (23). In BM hyperactivation of BAFF/APRIL has also been found (24), and in the spleen BAFF/APRIL can also increase long-lived plasma cells (25). This may account for some of the RTX treatment failures.

With the use of belimumab, there are continued case reports (26) of effective treatment of SLE-ITP.Sequential treatment of RTX and belimumab decreases the formation of long-lived plasma cells, enabling long-term disease management, offering a new approach for treating SLE-ITP (26). Belimumab is a recombinant humanized IgG2-λ monoclonal antibody, which can bind to BAFF to inhibit the differentiation, survival and antibody secretion of naive B lymphocytes, memory cells, marginal B lymphocytes, follicular B lymphocytes and plasma cells (27). However, because APRIL can still bind to TACI and BCMA, belimumab treatment is more commonly used to inhibit naive B lymphocytes (28), resulting in a relatively weak effect on plasma cells and a less rapid response to therapy for life-threatening thrombocytopenia.

In 2021, telitacicept was approved as a treatment for active SLE in China. It is a new type of fusion protein biologic that is not a traditional monoclonal antibody (18). It is fusion protein comprising a TACI (the common ligand of BLys and APRIL) receptor fused to the fragment crystallizable (Fc) domain of human IgG, which can simultaneously block the binding of BLyS and APRIL to TACI, inhibit plasma cell differentiation and survival, and affect the secretion of autoantibodies and pro-inflammatory cytokines (29). Because APRIL regulates the survival and function of long-lived plasma cells, it offers superior control of disease activity compared to belimumab and achieves multi-stage, multifaceted inhibition of B-cells.

With clinical application, telitacicept has demonstrated positive therapeutic outcomes. Wu et al. have reported that a phase 2b clinical trial of telitacicept in SLE demonstrated a notably higher percentage of patients achieving an SRI-4 response compared to the placebo group at week 48 (30). A group of 30 LN patients reported by Huang et al., with poor response or adverse reaction to conventional GCs showed a significant reduction in disease severity after receiving telitacicept treatment (31). Fan et al. described a case of lupus hepatitis in a patient with persistent liver damage despite treatment with various cDMARDs and belimumab. The patient’s liver function returned to normal after switching to telitacicept for one month (13). In addition to SLE, telitacicept has been tried for other immune system disorders, including primary sjögren’s syndrome, systemic vasculitis, neuromyelitis optica, MG, IgG4-associated diseases, and many other immune system disorders (5, 12, 32–34).

Our patient had persistent severely low platelets and a very high risk of bleeding, necessitating mega doses of MP to maintain platelets at a relatively safe level. Based on past experience, we introduced CsA and hetrombopag in the hope of raising platelets and helping MP decrease. However, the patient experienced recurrent severe drug-induced hepatitis. Inflammation, oxidative stress, and apoptosis for a variety of reasons (35), resulting in hepatic impairment frequently seen in treatment with DMARDs (36). Considering the drug-induced liver injury and time to take effect, we did not choose rituximab, belimumab, or other cDMARDs.Instead, we tried combining MP directly with telitacicept and achieved a positive therapeutic outcome. Platelet levels normalized rapidly without inducing liver damage, ensuring the patient’s safety.

## Conclusion

To our knowledge, this is the first case to report SLE-ITP treated with only GCs in combination with telitacicept. Given that severe thrombocytopenia can be life-threatening, the selection of effective drugs can significantly aid in achieving disease remission.This case suggests that telitacicept may be a new treatment option when conventional treatment for SLE-ITP is ineffective.

## Data Availability

The original contributions presented in the study are included in the article/supplementary material. Further inquiries can be directed to the corresponding author.
